# Sample-to-answer direct real-time PCR detection of *Anaplasma phagocytophilum*, *Ehrlichia* spp., and *Babesia* spp. infections in whole-blood specimens

**DOI:** 10.1128/spectrum.00655-24

**Published:** 2024-07-09

**Authors:** Georgia Colasante, Keana Makari, Tara I. Hummel, Caitlin Murphy

**Affiliations:** 1 HNL Lab Medicine, Allentown, Pennsylvania, USA; University of Maryland School of Medicine, Baltimore, Maryland, USA

**Keywords:** tick-borne infections, sample-to-answer, real-time PCR

## Abstract

Emerging tick-borne illnesses, such as anaplasmosis, babesiosis, or ehrlichiosis, are caused by obligate intracellular pathogens that have clinically comparable presentations. Diagnostics used in laboratories today are serologic assays and blood smear analyses, which have known diagnostic limits. This study evaluated the performance of a sample-to-answer direct real-time PCR laboratory-developed test for the multiplex qualitative detection of *Anaplasma*, *Babesia*, and *Ehrlichia* DNA in whole-blood specimens. Compared to two standard-of-care (SOC) methods, the DiaSorin tick-borne laboratory-developed test for *Anaplasma* detection demonstrated a positive percent agreement (PPA) and negative percent agreement (NPA) of 100% (95% CI, 0.80 to 1.0) and 89% (95% CI, 0.74 to 0.97), respectively with a discordant rate of 9.3% against microscopy. After discordant resolution, the NPA increased to 100%. For *Babesia*, the test demonstrated a PPA of 100% (95% CI, 0.90 to 1.0) and NPA of 100% (95% CI, 0.90 to 1.0). Compared to a SOC PCR method *Anaplasma* samples showed a PPA of 100% (95% CI, 0.66 to 1.0) and NPA of 100% (95% CI, 0.90 to 1.0). *Ehrlichia* results showed a PPA of 100% (95% CI, 0.69 to 1.0) and NPA of 100% (95% CI, 0.90 to 1.0). The total percent agreement was 98% (95% CI, 0.95 to 0.99) with a κ statistic of 0.95 (95% CI, 0.90 to 0.99) or almost perfect agreement compared to SOC methods. This laboratory-developed test for detecting *Anaplasma*, *Babesia*, and *Ehrlichia* DNA provides rapid and reliable detection of tick-borne infections without nucleic acid extraction.

**IMPORTANCE:**

This work demonstrates that detection of tick-borne illnesses, such as anaplasmosis, babesiosis, or ehrlichiosis, can be performed directly from whole blood with no extraction. The assay described here has a high positive and negative percent agreement with existing methods and is used as the standard of care. An increasing incidence of tick-borne illness combined with shortage of well-trained technologists to perform traditional manual testing, testing options that can be adapted to various lab settings, are of the utmost importance.

## INTRODUCTION

Ticks are the second most ecologically important vectors of pathogens that cause both human and animal diseases worldwide (1). Ticks are obligate hematophagous arachnids that parasitize every class of vertebrates in almost every region of the world. In the United States, ticks are the primary disease vector, responsible for 77%–95% of all cases of reported vector-borne disease yearly (2). They can transmit zoonotic pathogens, such as viruses, bacteria, protozoa, parasites, and toxins (1–5). Among these, *Borrelia* spp., which causes Lyme disease, is the most prevalent, followed by *Babesia* spp., *Anaplasma* spp., *Ehrlichia* spp., and *Rickettsia rickettsii* (1, 6–8). Transmission of these pathogens to humans and other animals is through salivary secretions, feces, coxal fluids, and regurgitation (6).

Tick-borne diseases are increasing in prevalence (9). This is likely due to environmental changes in combination with increased travel and mobility of humans and pets, and this has resulted in a rapid extension of the zoogeographical range for many tick species (10–12). Several testing methodologies are available in the clinical laboratory to aid in the diagnosis and patient management of tick-borne diseases. These methods include light microscopy for detecting organisms in tissues or peripheral blood, measurement of specific antibody responses, culture isolation, and molecularly based assays. The potential of the molecular diagnostic approach has been recognized: nucleic acid amplification testing (NAAT) can detect tick-borne pathogen DNA in multiple sample types, including blood, cerebrospinal fluid, synovial fluid, and biopsy tissues (1, 13, 14). Compared to NAAT‐based molecular assays, available serological tests generally have low specificity and sensitivity, particularly during the early phase of infection (1, 15, 16). Additionally, serologic tests provide significant logistical issues for many laboratories undertaking high-volume testing. These elements raise the possibility of incomplete reporting of these infections, inaccurate diagnoses, and delays in treatment. Despite the advantages, NAAT assays are still not always used to diagnose tick-borne pathogens, and products cleared by regulatory bodies are not available.

Nevertheless, many current NAAT‐based molecular detection methods for *Anaplasma*, *Ehrlichia*, and *Babesia* provide results after extracting DNA. Sample-to-answer assays have become popular and straightforward, bypassing the need for many of the pre-analytic sample preparation and post-amplification analysis requirements of a traditional molecular laboratory. This allows these tests to be performed with minimal hands-on time in settings without sophisticated expertise in molecular biology, including locations with limited resources.

Therefore, we aimed to evaluate the analytical and clinical performance of a sample-to-answer rapid real-time PCR method to detect anaplasmosis, babesiosis, or ehrlichiosis infections in whole-blood (WB) specimens without extraction. In addition, the study explored the possibility of using the DiaSorin Molecular 8-well Direct Amplification Disc together with the LIAISON MDX instrument as a part of the system for this laboratory-developed test (LDT) using commercially available Analyte Specific Reagents.

## MATERIALS AND METHODS

### Study design

Assay performance was evaluated using both prospective and retrospective specimens. EDTA-preserved WB samples were collected from patients for whom diagnostic tests (blood smear analysis and/or PCR) for human anaplasmosis, babesiosis, or ehrlichiosis were ordered by healthcare providers. After routine testing, specimens were aliquoted into 1.5-mL cryovial tubes and frozen at −80°C. Clinical testing was performed on 16 *Anaplasma*-positive, 36 *Babesia*-positive, and 10 *Ehrlichia*-positive specimens initially submitted for routine testing at HNL Lab Medicine. A total of 36 negative samples used for the study were submitted for blood smear analysis and PCR testing for diagnosis of tick-borne pathogens before calling a specimen negative. Retrospective samples were thawed, and prospective samples were aliquoted and subsequently tested using the DiaSorin tick-borne LDT. All residual samples were de-identified before enrollment in the study and testing.

### DiaSorin molecular Real-Time PCR Laboratory-Developed test

All WB specimens were prepared as a 1:1 dilution with phosphate-buffered saline (PBS) without magnesium and calcium (catalog # A1285601) (Gibco; Thermo Fisher Scientific, Inc., Waltham, MA, USA) and then tested using the DiaSorin tick-borne LDT. Amplification of *Anaplasma*, *Babesia*, and *Ehrlichia* was carried out on the DiaSorin Molecular 8-well Direct Amplification Disc (DAD) using the LIAISON MDX instrument. The LDT consisted of DiaSorin Molecular Analyte-Specific Reagent (ASR) primer pairs for Anaplasma (MOL9062), Babesia (MOL9063), Ehrlichia (MOL9064), plus reagents, and disc consumable (DiaSorin Molecular LLC, Cypress, CA, USA). All reactions were prepared immediately before amplification as reaction mix containing the following components: (i) 0.5 µL of *Anaplasma phagocytophilum* primer pair, (ii) 0.5 µL of *Babesia* spp. primer pair, and (iii) 0.5 µL of *Ehrlichia* spp. primer pair; (iv) 20 µL of TA master mix (MOL9070), (v) 1 µL of Simplexa Extraction and Amplification Control primer pair, (vi) 1 µL of Simplexa Extraction and Amplification Control DNA, and (vii) 26.5 µL of nuclease-free water.

Briefly, 50 µL of diluted sample or control was added to the “SAMPLE” well, and 50 µL of reaction mix was added to the “R” well. All testings were performed with the following parameters: Hold 1 cycle of 97°C for 300 s at 5 ramp rate (C/s); mixing at 97°C for 120 s; 40 cycle count; denaturation at 97°C for 10 s at 5 ramp rate (C/s); annealing at 60°C for 30 s at 5 ramp rate (C/s). Targets and internal control fluorescence thresholds were set at 5,000. Data collection and analysis were performed with LIAISON MDX Studio software. The cycle threshold (Ct) value for positive samples was defined as the cycle number at which the fluorescence generated within a reaction crossed the fluorescence threshold.

### Standard of care methods (SOC)

The SOC methods used for the assessment were microscopy and real-time PCR testing. Initial testing by the SOC methods was performed, and the resulting interpretation was used to enroll positive and negative *Anaplasma*, *Babesia*, and *Ehrlichia* specimens into the study. For SOC 1 (microscopy), blood smear slides were made from whole-blood samples, stained by Wright’s method, and examined microscopically under oil immersion for leukocytic intracellular morulae by trained clinical staff. For SOC 2 (multiplex real-time PCR LDT method), 5 µL of extracted DNA was added to 5 µL of master mix that contained 0.2 µL of *Anaplasma* primer pair (MOL9062), 0.2 µL of *Ehrlichia* primer pair (MOL9064), 4 µL of TA master mix (MOL9070), 0.2 µL of Simplexa Extraction and Amplification Control primer pair (MOL9000), and 0.4 µL of nuclease-free water. The amplification reactions using DiaSorin Molecular *Anaplasma* and *Ehrlichia* primer pairs were carried out on a 96-well Universal Disc using the LIAISON MDX instrument with dye detection on for CRF610 (*Ehrlichia*), FAM (*Anaplasma*), and Q670 (Internal control). Data collection and analysis were performed with LIAISON MDX Studio software. The following cycling conditions were used: 1 cycle at 97°C for 120 s followed by 40 cycles at 97°C for 10 s with a ramp speed of 2°C/s, and 60°C for 30 s with a ramp speed of 2°C /s with capture mode on. The test time of the SOC 2 *Anaplasma/Ehrlichia* real-time PCR LDT assay from sample processing to result is approximately 60 min.

### Analytical performance

The limit of detection (LoD) was determined using quantified stocks of *Anaplasma* (Exact Diagnostics, Fort Worth, TX, USA), *Ehrlichia* (Exact Diagnostics, Fort Worth, TX, USA), and *Babesia* (ATCC PRA-398DQ) serially diluted in a negative pooled human whole-blood matrix. The LoD was determined by two methods: positive rate and Probit analyses. The positive rate was defined as the lowest dilution at which all replicates resulted in *Anaplasma*, *Babesia*, or *Ehrlichia* positive with a 100% detection rate. The LoD by Probit was determined as the lowest detectable dilution at which the quantified *Anaplasma*, *Babesia*, or *Ehrlichia* stocks (copies/mL) resulted positive with a 95% probability of detection.

### Resolution of discordant results

Results were considered discordant when the DiaSorin Molecular LDT assay did not agree qualitatively with the SOC results. In such cases, molecular testing was repeated for the discordant assay when a remnant sample was available, and samples were sent to a reference laboratory for PCR testing.

### Statistical methods

Percent sensitivity, specificity, Kappa, Probit, and two-sided (upper/lower) 95% confidence interval (CI) were calculated using Microsoft Office Excel 365 MSO software (Microsoft, Redmond, WA). The sensitivity was calculated as TP/(TP +FN) × 100, and the specificity was calculated as TN/(TN +FP) × 100, where TP is true positive, FN is false negative, TN is true negative, and FP is false positive. Cohen’s kappa values (κ) were also calculated as a measure of overall agreement, with values categorized as almost-perfect (>0.90), strong (0.80 to 0.90), moderate (0.60 to 0.79), weak (0.40 to 0.59), minimal (0.21 to 0.39), or none (0 to 0.20) (17, 18). Probit analyses were used for the copies/mL determination of the analytical sensitivity study. The dose–response 95th percentile (with 95% CI) model was assessed using the Finney and Stevens calculations (19). The discordance rate was calculated as (FP + FN)/total number of samples tested × 100. The analysis of variance (ANOVA) on the Ct values was performed using GraphPad Prism version 10 for Mac (GraphPad Software, San Diego, California USA, www.graphpad.com).

## RESULTS

Following testing of specimens, the DiaSorin Tick-borne LDT and the SOCs showed a total percent agreement of 98% (95% CI, 0.95 to 0.99), with a κ statistic of 0.95 (95% CI, 0.90 to 0.99), demonstrating almost-perfect agreement between methods for three analytes. All analytes had a positive percent agreement of 100% when tested using the DiaSorin Ticks LDT (Table 1). A negative percent agreement of 89% (95% CI, 0.74 to 0.97) was observed for the *Anaplasma* target, while *Babesia* showed a negative percent agreement of 100% against SOC 1. Four clinical specimens for *Anaplasma* were positive on the DiaSorin Ticks LDT and were identified as negative by SOC 1. An overall discordance rate of 9.3% was found for *Anaplasma* between DiaSorin Tick-borne LDT and SOC 1. A positive and negative percent agreement of 100% was observed for a second set of *Anaplasma* samples and *Ehrlichia* targets when tested against SOC 2 (Table 1).

**TABLE 1:** Clinical performance comparison between the laboratory standard of care methods vs. DiaSorin Tick-borne LDT

DiaSorin Tick-borne LDT	SOC 1^*a*^		(± 95% CI)^*b*^^,*c*^			Discordance rate
	Positive	Negative	PPA^*d*^	NPA^*e*^	Kappa^*f*^	
*Anaplasma*						
Positive	7	4^*g*^	100%	89%	0.72	9.3%
Negative	0	32	(0.59–1.0)	(0.74–0.97)	(0.47–0.97)	
*Babesia*						
Positive	36	0	100%	100%	1	0.0%
Negative	0	36	(0.90–1.0)	(0.90–1.0)	(0.99–1.0)	

DiaSorin Tick-borne LDT	SOC 2^*h*^		(± 95% CI)^*b*^^,*c*^			Discordance Rate
	Positive	Negative	PPA^*d*^	NPA^*e*^	Kappa^*f*^	
*Anaplasma*						
Positive	9	0	100%	100%	1	0.0%
Negative	0	36	(0.66–1.0)	(0.90–1.0)	(0.99–1.0)	
*Ehrlichia*						
Positive	10	0	100%	100%	1	0.0%
Negative	0	36	(0.69–1.0)	(0.90–1.0)	(0.99–1.0)	

^*a*^ Standard of care method 1 (blood-smear microscopy).

^*b*^ ±, Upper/lower 95%.

^*c*^ CI, confidence interval.

^*d*^ Positive Percent Agreement (PPA).

^*e*^ Negative Percent Agreement (NPA).

^*f*^ Almost perfect (>0.90), strong (0.80 to 0.90), moderate (0.60 to 0.79), weak (0.40 to 0.59), minimal (0.21 to 0.39), or none (0 to 0.20).

^*g*^ Four samples had Ct of 28.3, 29.5, 32.2, and 33.3 by the DiaSorin Tick-borne LDT.

^*h*^ Standard of care method 2 (real-time PCR LDT).

Four specimens (Ana-09, Ana-15, Ana-16, Ana-20) that were negative by standard-of-care 1 (blood-smear microscopy) were identified as positive by the DiaSorin Tick-borne LDT. The four samples had Ct values of 28.3, 29.5, 32.2, and 33.3 for *Anaplasma*, respectively. All samples were sent to a reference lab for PCR testing. The results revealed that all samples were positive by PCR. Therefore, these samples were categorized as true positive during discordant resolution. Following discordant analysis, the DiaSorin Tick-borne LDT assay showed an improvement of NPA to 100% (95% CI, 0.90 to 1.0). Furthermore, the total percent agreement of DiaSorin Tick-borne LDT and SOC 1 increased to 100% (95% CI, 0.90 to 1.0), with a κ statistic of 1.0 (95% CI, 0.99 to 1.0). The results are summarized in Table 2.

**TABLE 2:** Details of *Anaplasma* discordant sample analysis

Sample ID	SOC 1^*a*^	DiaSorin Tick-borne LDT Ct^*b*^	Comments
Ana-09	NEG	28.3	*Anaplasma* PCR POS (Ref. lab^*c*^)
Ana-15	NEG	29.5	*Anaplasma* PCR POS (Ref. lab)
Ana-16	NEG	32.2	*Anaplasma* PCR POS (Ref. lab)
Ana-20	NEG	33.3	*Anaplasma* PCR POS (Ref. lab)

^*a*^ Standard of care 1 (blood-smear microscopy).

^*b*^ Ct, cycle threshold.

^*c*^ Reference laboratory PCR sent out.

The LoD was defined as the minimum concentration with detection values of 100% by positive rate and 95% by Probit analysis. The LoD established by percent positive rate was 62.5 copies/mL for all three analytes. The LoD results were further subjected to Probit analysis. The 95% detection limit value for *Anaplasma* was 47 ±  26 copies/mL; for *Babesia*, 43 ±  13 copies/mL; and for *Ehrlichia*, 41 ±  08 copies/mL (Table 3).

**TABLE 3:** Summary of limit of detection results for the DiaSorin Tick-borne LDT

Analytes	Positive rate %				Probit (± 95% CI)^*a*^^,*b*^	
	copies/mL	copies/Rx	% Detection	Mean Ct ±SD [% coefficient of variation (CV)]	copies/mL	copies/Rx
*Anaplasma*	62.5	6	100% (10/10)	36.7 ± 0.9 (2.4%)	47 ± 26	5
*Babesia*	62.5	6	100% (10/10)	32.6 ± 0.4 (1.2%)	43 ± 13	4
*Ehrlichia*	62.5	6	100% (10/10)	34.5 ± 0.9 (2.7%)	41 ± 08	4

^*a*^ CI, confidence interval.

^*b*^ ±, Upper/lower 95%.

The inter-and intra-assay reproducibility was performed using three replicates (*n* = 3) of each point of dilutions tested between 9 × 10^3^ copies/mL and 5 × 10^2^ copies/mL using contrived samples of *Anaplasma*, *Babesia*, and *Ehrlichia*. Testing was conducted over five days. The means ± standard deviation (SD), Ct values, and coefficients of variation (CV) are shown in Table 4. The CV for the inter-assay assessment ranged from 1.0% to 3.0 % for *Anaplasma*, 2.9% to 4.6 % for *Babesia*, and 2.0% to 2.9 % for *Ehrlichia*. A one-way ANOVA was used to compare the mean Ct of each of the three analytes (*Anaplasma*, *Babesia*, and *Ehrlichia*) during the course of the reproducibility testing, and the *P*-value was determined to be 0.5060. This difference is considered to be not statistically significant demonstrating a high degree of assay reproducibility.

**TABLE 4:** Summary of inter- and intra-assay reproducibility of the *Anaplasma*, *Babesia*, and *Ehrlichia* targets tested using the DiaSorin Tick-borne LDT

Analytes	Concentration (copies/mL)	Mean Ct ±SD (% CV)^*a*^	
		Inter-assay reproducibility	Intra-assay reproducibility
*Anaplasma*	9,000	29.0 ± 0.3 (1.0)	30.7 ± 0.2 (0.7)
	1,000	31.0 ± 0.9 (2.8)	31.0 ± 0.2 (0.5)
	500	32.0 ± 1.0 (3.0)	32.0 ± 0.2 (0.8)
*Babesia*	9,000	27.0 ± 0.8 (2.9)	27.0 ± 0.5 (1.7)
	1,000	29.8 ± 0.9 (3.0)	29.8 ± 0.2 (0.7)
	500	31.0 ± 1.4 (4.6)	31.0 ± 0.3 (0.9)
*Ehrlichia*	9,000	26.3 ± 0.8 (2.9)	26.3 ± 0.4 (1.5)
	1,000	29.4 ± 0.6 (2.2)	29.4 ± 0.2 (0.7)
	500	30.9 ± 0.6 (2.0)	31.0 ± 0.6 (1.9)

^*a*^ Ct, SD, and % CV.

A high degree of intra-assay precision was also observed based on the analysis. A one-way ANOVA was conducted in the same fashion as the inter-assay assessment and yielded a *P*-value of 0.3248; no significant differences among replicates were evident between analytes (Table 4). Therefore, the precision and reproducibility of the DiaSorin Tick-borne LDT were excellent, and all analytes were reliably and accurately detected.

**Fig 1:**
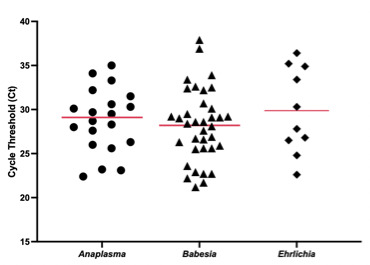
Distribution of Ct values from all positive *Anaplasma*, *Babesia*, and *Ehrlichia* specimens. Solid red lines indicate average Ct.

A total of 66 positive samples were tested using the DiaSorin Tick-borne LDT assay. The Ct value distribution obtained by the assay from tick-infection positive patients is shown in Fig. 1. Across all three targets, the Ct values for the positive samples were quite comparable; *Anaplasma* (mean, 28.8; median, 29.1; range, 22.4 to 35), *Babesia* (mean, 28.2; median, 28.5; range, 21.2 to 37.9), and *Ehrlichia* (mean, 29.9; median, 29.1; range, 22.6 to 36.4). The positive samples showed an average Ct value of 28.9, with a range of 21.2 to 37.9 Cts for all targets. Samples were both prospective and retrospective.

## DISCUSSION

Blood parasite smears have remained the gold standard for the diagnosis of parasitic diseases, but as the incidence and awareness of tick-borne infections continue to increase, newer methods of diagnosis are needed. Real-time PCR-based molecular assays are highly effective during the acute phase of infection and are increasingly replacing or supplementing blood smears and/or serological methods for diagnosis.

A challenge of both traditional microscopic methods and some molecular methods is the need for trained personnel in complex, time-consuming techniques. This is becoming more of a challenge in a workforce struggling to find trained laboratory scientists. In this study, we aimed to simplify the detection of blood-borne parasites using a sample-to-answer whole-blood PCR technique with no need for separate nucleic acid extraction using the DiaSorin Molecular PCR platform. The study objective was twofold: to determine if appropriate analytical sensitivity and specificity could be reached in whole blood without an extraction step and optimize testing in an eight-well disc format for adoption in the clinical laboratory. Currently, most lab-developed testing using the DiaSorin ASR primers and system is performed in a 96-well disc format.

Three commonly encountered tick-borne infections in our patient population are those caused by *Babesia*, *Anaplasma*, and *Ehrlichia*. These infections have been increasing in incidence, causing symptomology ranging from subclinical to those requiring hospitalization (20–22). During this study, we analyzed 98 samples. These samples were submitted for SOC testing to our laboratory or to other labs for suspicion of a tick-borne or other blood parasite infection. The results of the sample-to-answer PCR assay were compared to microscopic methods or molecular detection performed at different laboratories with validated testing methodologies. Following discordant analysis with an alternative testing method, the overall NPA and PPA for all targets was 100%.

Optimization for the first time of the eight-well Direct Amplification Disc format for non-extracted whole-blood specimens and the simultaneous multiplex detection of *Anaplasma*, *Babesia*, and *Ehrlichia* targets required a simple 1:1 dilution with PBS (without magnesium and calcium). This simple dilution step was used to prevent inhibition-related internal control errors. Nonetheless, the real-time PCR assay’s reproducibility and sensitivity were unaffected by the dilution requirement. Intra- and inter-assay reproducibility and the relationship between parasite loads (Ct values) from tick-borne-positive samples in whole-blood specimens were examined. Even though the samples require a simple 1:1 dilution, the study showed high reproducibility with low CVs for all targets (Table 4). It should be noted that the internal control exhibited a consistent average of 28 Cts despite the significant proportion of substances in whole-blood specimens that may inhibit PCR (data not shown).

Furthermore, the DiaSorin Tick-borne LDT assay is also helpful in cases where organisms may be difficult to detect by less sensitive methods. The analytical sensitivity of the test was demonstrated to be high, with the ability to detect between four and five copies of organisms’ target DNA (Table 3). The assay’s ability to identify infections of lower bacteremia that are undetectable by microscopic examination was highlighted in our study by the four discrepant specimens for *Anaplasma*. These samples showed a range of 28.3 to 33.3 Cts. Based on the Ct value distribution found in most clinical samples in Fig. 1, it can be theorized that these patients had low levels of bacteremia. Typically, *Anaplasma* and *Ehrlichia* can be diagnosed through blood smears by identifying cytoplasmic inclusions (morulae). However, blood smears are not considered a gold standard diagnostic tool for *Anaplasma* or *Ehrlichia* due to the low sensitivity, mainly depending on the stage of infection. Studies have shown that the maximum positivity for anaplasmosis is obtained in the early phase of illness when morulae are likely to be visible in blood smear analysis and before patients have developed an effective antibody response (23–25). While these four discrepant specimens were negative by the ordered SOC 1 microscopic evaluation, they were positive by PCR at multiple laboratories. Even when the slides were examined in the context of being known positives, no inclusions suggestive of *Anaplasma* were identified. Discrepant results were likely not seen with *Ehrlichia* as this is uncommon in the lab’s geographical coverage area, and positive samples for testing were shared from a lab where the incidence of *Ehrlichia* was much higher. Similarly, identifying *Babesia* is more straightforward than *Anaplasma* or *Ehrlichia* since infection by the organism can be identified by rings inside red cells when evaluating patient blood smears. Optimal and accurate diagnosis of *Babesia* from blood smears depends on the minimal lag time between sample collection and the preparation of the blood smears. Microscopic identification may also be impacted by prior treatment. These factors have a less profound impact on more sensitive diagnostic molecular methods.

Molecular testing of tickborne pathogens is beneficial because identifying morulae in particular cell types cannot be used for definitive detection of *Anaplasma* versus *Ehrlichia*. Differentiation may not be critical for treatment, but it can have implications for public health and epidemiology. Additionally, *Ixodes scapularis*, the vector for *Borrelia burgdorferi*, *A. phagocytophilum*, and *Babesia microti*, can have incidences of almost 25% coinfection with multiple pathogens (21). Coinfections with vector-borne pathogens can complicate diagnosis and treatment since they can cause more severe clinical symptoms and alter clinical illness presentations that are normally associated with individual infections (26–28). The possibility of transmission of coinfection in humans and minimal knowledge regarding the outlook of polymicrobial infections suggests combination testing would benefit high-risk individuals.

A noted limitation of this study was the need for species-specific information on the *Babesia* and *Ehrlichia* detected in the samples. The ASR primer pairs used for this study from DiaSorin were designed to target a highly conserved genomic region of each organism and should detect infections caused by the various circulating species of *Babesia* and *Ehrlichia*, but further in-depth study across a broader geographic range would be needed to demonstrate NPA/PPA in a more robust sample set. Studies and *in silico* analysis show that the primer pairs can detect *A. phagocytophilum*, *Babesia* species including *microti*, *duncani*, and *divergens*, and *Ehrlichia* species including species of *chaffeensis*, *muris*, *ewingii*, and *canis*. Additionally, studies have indicated that the primers do not share significant homology with other pathogens, including those that can have overlapping symptomology. Details and additional information are available from DiaSorin Technical Assistance.

Despite this limitation, this study was able to demonstrate that using whole blood samples, coupled with the use of the DiaSorin 8-well disc, simple and reliable results for the detection of blood-borne parasites could be achieved. With the eight-well Direct Amplification Disc (DAD)’s multi-use capabilities and DiaSorin’s direct chemistry, the DiaSorin Tick-borne LDT employs a sample-to-answer format that produces results for up to eight samples in 1 h and requires just dilution-only sample preparation instead of formal nucleic acid extraction. The assay can be run on-demand without batching, which is a feature that can help laboratories without the resources to operate off-board extraction instruments, allowing for the introduction of molecular detection of *Babesia*, *Anaplasma*, and *Ehrlichia* in a cost-effective manner for accurate and timely diagnosis.
